# Scanner and kVp dependence of measured CT numbers in the ACR CT phantom

**DOI:** 10.1120/jacmp.v14i6.4417

**Published:** 2013-11-04

**Authors:** Robert J. Cropp, Petar Seslija, David Tso, Yogesh Thakur

**Affiliations:** ^1^ Integrated Medical Imaging Vancouver Coastal Health Vancouver British Columbia Canada; ^2^ Department of Radiology Faculty of Medicine University of British Columbia Vancouver British Columbia Canada

**Keywords:** CT number, ACR phantom, CT quality control

## Abstract

Quality control testing of CT scanners in our region includes a measurement of CT numbers in the American College of Radiology (ACR) CT phantom using a standardized protocol. CT number values are clinically relevant in determining the composition of various tissues in the body. Accuracy is important in the characterization of tumors, assessment of coronary calcium, and identification of urinary stone composition. Effective quality control requires that tolerance ranges of CT number values be defined: a measured value outside the range indicates the need for further investigation and possible recalibration of the scanner. This paper presents the results of CT number measurements on 36 scanners (25 GE, 10 Siemens and 1 Toshiba) at each available kVp. Among the five materials (solid water, air, polyethylene, acrylic, bone‐equivalent) the measured CT numbers exhibit manufacturer and kVp dependence, which should be taken into account when defining tolerances. With this scan protocol, air and solid water values are significantly higher on GE scanners than on Siemens scanners (p‐value<0.01 at each kVp). The CT numbers of polyethylene and acrylic increase with kVp, while the bone‐equivalent CT number decreases. These results are used to define manufacturer‐ and kVp‐specific tolerance ranges for the CT numbers of each material in this phantom, which will be used in our quality control program.

PACS numbers: 87.57.qp, 87.57.C‐

## I. INTRODUCTION

Computed Tomography (CT) is a vital diagnostic imaging modality in medical practice. Technological improvements in multidetector CT (MDCT) have led to lower dose protocols with similar or improved image quality. Further technological improvements have increased the number of CT scans performed per year.^1^, ^2^, ^3^ In our health authority's region, CT scans have increased from 275,000 to 300,000 per annum between 2010 and 2012.

At equipment commissioning, medical physicists or other qualified personnel perform a variety of tests to ensure scanner operation is within manufacturer specification and to establish baseline values for equipment performance. Baseline values are monitored for the lifetime of the equipment to ensure performance degradation is identified, and addressed, before potential negative impact to patient care. A well‐established quality control (QC) program will include monitoring of vital image quality metrics to ensure scanner performance has not changed. For CT, these metrics include: low‐contrast detectability, high‐contrast (spatial) resolution, modulation transfer function, CT number uniformity, and CT number calibration.^(^ ^4^ ^,^ ^5^ ^)^

The CT number of a material at a given kVp is defined by its linear attenuation coefficient μ. and the attenuation coefficient of water at the same kVp:

(1) $$\text{CT}\;\text{number}=1000\times (\mu -\mu_{\text{water}})/\mu_{\text{water}}$$

The unit for CT numbers is Hounsfield units (HU); from the definition, water is zero HU and air is −1000HU. CT manufacturers typically release specifications on tolerance for CT numbers. For example, GE specifies a mean water value within ±3HU of zero when using a specific quality assurance protocol, which is specified in the scanner's technical reference manual. Regulatory tolerances may also be defined in some jurisdictions; for example, Health Canada's Safety Code 35 states that water values must be within ±4HU of zero and air values must be within ±10HUof−1000.^(4)^ The European Guidelines on Quality Criteria also state a ±4HU tolerance for water and a ±5HU tolerance for CT number accuracy from baseline measurements for a given material.^(5)^

Manufacturer‐supplied phantoms are typically used for scanner calibration and to monitor equipment performance for a given scanner make and model. Other phantoms, such as the Catphan (The Phantom Laboratory, Salem, NY) or ACR Accreditation Phantom (model 464, Gammex, Middleton, WI) are typically used by physicists to monitor image quality on various scanners. The ACR phantom is also endorsed by the American College of Radiology for CT accreditation in the United States.

In 2011, our physics group's responsibility in our health region tripled due to a consolidation of medical imaging services across multiple publically funded health authorities. The ACR phantom was used by the physics group to establish baseline values for all scanners under the group's responsibility. For CT number accuracy, the ACR recommends that scans are performed between 120 and 130 kVp, and gives CT number ranges which are based on the “average values obtained from multiple scanner models”.^(6)^ However, with the recent trend towards BMI‐based tube potential modulation, specifically in chest examinations,^(7)^ and the use of higher kVps to penetrate the posterior fossa in routine head examinations, the physics group decided to measure CT accuracy at all available kVps. A wide range of kVps can be expected in clinical use since kVp can be optimized based on application and patient size.^(8)^ For materials other than water and air, some dependence of CT number on kVp is expected, primarily because of the photoelectric interaction's strong dependence on both photon energy and atomic number. This causes a given material's attenuation coefficient to depend differently on energy than water's coefficient, leading to a dependence of the material's CT number on kVp.

Accurate CT number measurements are relevant in several clinical applications, which rely on quantitative HU values for diagnosis. When making quantitative measurements, the limits on achievable accuracy, and the variation between scanners, should be taken into account. QC data from a standard phantom may be useful in characterizing the accuracy and intrascanner variation of CT number measurements.

CT has been a useful tool in determining the extent of coronary atherosclerosis. CT evaluation of coronary arterial calcium has been established as providing both diagnostic and prognostic information with regards to atherosclerotic disease.^(^ ^9^ ^,^ ^10^ ^)^ The Agatston score, a commonly used clinical measurement, uses HU thresholds to determine the degree of calcification of coronary plaques (i.e.,Score1=130−199HU,Score2=200−299HU,Score3=300−399HU,Score4=>400HU).^(11)^ Nelson et al.^(12)^ demonstrated differences in phantom CT number values of up to 17.1 HU between CT scanners of different manufacturers. Of the 9553 subjects assessed for coronary calcium score, 336 or 3.5% of participants moved clinical Agatston score as a result of image calibration.

HU values also play a role in the quantitative evaluation of adrenal masses. A majority of adrenal adenomas are lipid rich and will have low attenuation on an unenhanced CT scan and rapidly wash out contrast on delayed scans.^(13)^ If the lesion has a density of 10 HU or below, it is likely an adenoma and no further workup is warranted.^(13)^ A biopsy is recommended if the adrenal lesion is greater than 10 HU on unenhanced CT and there is less than 60% contrast washout on the delayed scan.^(13)^

CT is the major method in characterizing renal cysts, which can be assessed using both unenhanced and contrast enhanced CT.^(14)^ Increased contrast uptake of the renal cyst postcontrast may be suggestive of a pathological process. Specifically, greater than 15 HU increase postcontrast would be suggestive of a malignancy, and cysts with changes less than 10 HU are usually considered benign.^(14)^ HU thresholds have been reported in the literature in differentiating renal angiomyolipomas (AML) from renal cell carcinoma where CT numbers of −10HU or lower are suggestive of AML on nonenhanced CT.^(15)^

HU differentiation is utilized in the characterization of urinary stone composition. Although the unenhanced CT scan is the gold standard for the diagnosis of urinary stones, the various types of stones overlap in radiodensities to preclude accurate material composition.^(16)^ Dual energy CT enables simultaneous low‐ and high‐energy scanning, which allows one to determine the composition of materials with similar electron density but varying photon absorption, such as calcium and uric acid.^(17)^ The composition of urinary stones can be accurately identified using the attenuation ratio at the two energy levels.^(18)^ Inaccurate CT numbers may result in incorrect material identification of urinary stones.

Taking into consideration the clinical applications of CT number measurements over different ranges of HU values and the potential use of various kVps, we present results from our QC program on the five material inserts in the ACR phantom at multiple kVps. Our results show that both kVp and scanner manufacturer can influence the CT number accuracy. Using a standardized protocol, a new set of pass/fail criteria for specific scanners at multiple kVps is suggested.

## II. MATERIALS AND METHODS

There are a total of 36 CT scanners in our quality control program: 25 GE (GE Healthcare, Waukesha, WI), 10 Siemens (Siemens Healthcare, Erlangen, Germany) and 1 Toshiba (Toshiba Medical Systems, Tochigi‐ken, Japan). Table 1 lists all models.

Our QC program uses the ACR accreditation phantom for CT number measurements. Module 1 of this phantom contains five material inserts for this purpose: a water‐equivalent solid (henceforth referred to as solid water), air, polyethylene, acrylic, and bone‐equivalent (henceforth referred to as “bone”). Table 2 lists the recommended CT number ranges of these materials at 120–130 kVp, as given in the ACR accreditation instructions^(19)^

**Table 1:** Manufacturers and models of CT scanners in the quality control program. (The Siemens Symbia models are SPECT‐CT scanners.)

*Manufacturer*	*Model*	*Number of Units*
GE	LightSpeed VCT 64	17
	LightSpeed Pro	3
	CT750 HD	2
	LightSpeed Pro 32	1
	BrightSpeed	1
	LightSpeed Ultra	1
Siemens	Definition Flash	3
	Sensation	3
	Definition AS	1
	Symbia T6	2
	Symbia T2	1
Toshiba	Aquilion ONE	1

QC scans of the ACR phantom are performed on each scanner at a regular interval of six months, as set forth by our jurisdiction's regulatory body.^(20)^ The QC program incorporates all metrics required for ACR accreditation (low‐contrast detectability, slice width, uniformity, high‐contrast detectability, artifacts, and CT number accuracy). With the exception of CT number accuracy, all other metrics comply with ACR accreditation guidelines. The scan protocol for CT number measurements is standardized as much as possible, given the variety of scanner models. The phantom, resting on the base, is placed on the table and aligned to the system lasers. Table 3 lists the scan parameters.

Immediately after a scan is completed, the solid water CT number at each kVp is measured at the scanner console. If the value is outside ±5HU at any kVp, then the manufacturer‐supplied water phantom is scanned to verify the calibration. If the water value subsequently fails to meet the manufacturer specification (typically ±3HU for GE, ±4HU for Siemens), then the scanner is flagged for recalibration. The criterion for performing the manufacturer water phantom scan was originally more stringent (solid water value outside ±4HU, based on Safety Code 35 requirements^(4)^). However, we found that scanners often give values outside ±4HU with solid water, while still being within manufacturer specifications with the water phantom. Therefore, to avoid unnecessary water phantom scans, the criterion was relaxed to ±5HU, which is the tolerance stated in older (e.g., 2004) versions of the ACR phantom instructions. The current (2012) version of the ACR phantom instructions uses a wider tolerance of ±7HU, as shown in Table 2.

The full set of measurements is done off‐line. For each kVp, CT numbers are measured in a slice near the longitudinal center of the insert. The circular region‐of‐interest (ROI) tool in the image‐viewing application (Merge eFilm, Philips iSite) is used to define an ROI of approximately 2 cm^2^, and the mean CT number is recorded and compared to the recommended range.

**Figure 1:**
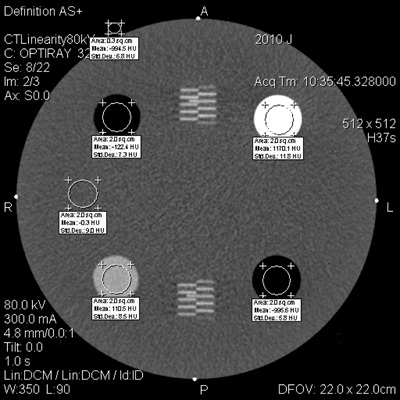
An image of Module 1 of the ACR phantom, showing the regions of interest (ROIs) for CT number measurements. The air ROI outside the phantom is smaller in order to fit inside the DFOV.

For this study only, air CT numbers were also measured in a second ROI outside the phantom. This was done because the measured air values inside the phantom insert were noticeably high on the GE and Toshiba scanners, usually at least 20 HU above the nominal value of −1000HU. If air values measured outside the phantom are systematically closer to −1000HU, then measuring outside may be a more reliable method for QC. The ROI outside the phantom is located anterior to the polyethylene insert and must be small enough to fit within the display field of view (DFOV); the ROI is typically about 0.3 cm^2^. Figure 1 illustrates the location of each ROI.

**Table 2:** Insert materials in the ACR CT accreditation phantom, and their ACR‐recommended CT number calibration criteria

*Insert Material*	*Recommended CT Number Range*
Water‐equivalent	−7to+7HU
Air	−1005to−970HU
Polyethylene	−107to−84HU
Acrylic	110 to 135 HU
Bone‐equivalent	850 to 970 HU

**Table 3:** Parameters for QC CT number measurement scans. The Siemens SPECT‐CT scanners are an exception — they use a body SFOV and kernel, instead

*Scan Parameter*	*Setting*
Scan type	Axial
Scan field of view size	Head
Beam width	Largest available
Rotation time	1 second
Tube current	300 mA (no modulation)
kVp	Repeat at each available kVp
Slice width	5 mm (or nearest available)
Display field of view	22 cm
Kernel (i.e., filter)	Default for the unit's standard head protocol

Our group uses two ACR phantoms for quality control testing. To verify that the two phantoms produce similar CT numbers, both phantoms were scanned on a single CT unit (a GE LightSpeed VCT) using identical setups, acquisition, and reconstruction parameters, at all available kVp settings. The mean CT number of each material within the ACR module was measured for both phantoms in all of the acquired scans. The CT number of each material was compared between scans of each phantom acquired at each kVp setting to determine if variations in manufacturing or material properties could account for any observed differences in our QC data.

## III. RESULTS

### A. Comparison of two ACR phantoms

Over all kVp settings, the maximum differences in mean CT number between the two phantoms were: 1.9 HU for water, 0.7 HU for air inside the phantom, 2.3 HU for polyethylene, 5 HU for acrylic, and 4.6 HU for bone.

### B. Overview of Scanner Results

Of the 36 scanners tested, one failed the water phantom tolerance. It was subsequently recalibrated and passed a water phantom test, but has not yet had another ACR phantom scan. Its CT numbers from before the recalibration are inaccurate and are not included in the results here.

Tables 4, 5, 6, 7, 8 present a summary of the measured CT numbers at four kVp values. Scanners are grouped by manufacturer: the tables cover 24 GE, seven Siemens, three Siemens SPECT‐CT, and one Toshiba scanner. The three SPECT‐CT scanners are separated from the other Siemens scanners because they use different kVp values; their CT numbers also differ from the others in some materials.

For the two larger groups of scanners (GE and Siemens non‐SPECT‐CT), the tables list interquartile range in addition to median, in order to characterize the spread in values. Interquartile range (IQR) is the difference between the first and third quartile values. These robust statistics are used instead of mean and standard deviation in order to reduce the influence of outliers, since the number of scanners is small. The median of the three SPECT‐CT scanners and the individual value from the one Toshiba scanner are also included for comparison.

The GE and Siemens non‐SPECT‐CT groups are not further subdivided by model because the limited number of scanners would make it difficult to draw conclusions in most cases. As an example, the three Siemens Definition Flash scanners were compared to the four Siemens Sensation and Definition scanners, using a t‐test. No p‐values were below 0.05 for any material or kVp.

**Table 4:** Solid water CT number measurements in HU. Data are from 24 GE, 7 Siemens, 3 Siemens SPECT‐CT, and 1 Toshiba scanners. For each entry, the first value is the median and the second value (in parentheses) is the interquartile range

	*80 kVp*	*100/110* ^a^ *kVp*	*120/130* ^a^ *kVp*	*135* ^b^ */140 kVp*
GE	3.4 (1.9)	4.3 (1.3)	5.1 (2.1)	5.8 (2.2)
Siemens	0.2 (2.5)	0.4 (1.5)	0.4 (2.0)	0.9 (2.1)
Siemens SPECT‐CT	−0.5 (‐)	−0.4 (‐)	−0.5 (‐)	−
Toshiba	−1.1 (‐)	0.0 (‐)	0.7 (‐)	0.8 (‐)

a The available kVp values on the Siemens SPECT‐CT scanners are 80, 110, and 130 kVp.

b The Toshiba scanner uses 135 kVp instead of 140.

**Table 5:** Air CT number measurements in HU. Data are from 24 GE, 7 Siemens, 3 Siemens SPECT‐CT, and 1 Toshiba scanners. For each group of scanners, air measurements both inside and outside the phantom are listed. For each entry, the first value is the median and the second value (in parentheses) is the interquartile range

	*80 kVp*	*100/10* ^a^ *kVp*	*120/130* ^a^ *kVp*	*135* ^b^ */140 kVp*
GE, inside	−973 (2.9)	−976 (1.6)	−977 (1.7)	−977 (1.7)
GE, outside	−991 (5.5)	−991 (2.8)	−990 (3.2)	−990 (2.5)
Siemens, inside	−995 (16.4)	−997 (15.2)	−996 (13.6)	−998 (12.3)
Siemens, outside	−997 (4.5)	−998 (3.5)	−999 (3.5)	−1000 (5.5)
Siemens SPECT‐CT, inside	−994 (‐)	−993 (‐)	−992 (‐)	−
Siemens SPECT‐CT, outside	−996 (‐)	−996 (‐)	−996 (‐)	−
Toshiba, inside	−971 (‐)	−975 (‐)	−974 (‐)	−977 (‐)
Toshiba, outside	−978 (‐)	−981 (‐)	−982 (‐)	−985 (‐)

a The available kVp values on the Siemens SPECT‐CT scanners are 80, 110, and 130 kVp.

b The Toshiba scanner uses 135 kVp instead of 140.

**Table 6:** Polyethylene CT number measurements in HU. Data are from 24 GE, 7 Siemens, 3 Siemens SPECT‐CT, and 1 Toshiba scanners. For each entry, the first value is the median and the second value (in parentheses) is the interquartile range

	*80 kVp*	*100/110* ^a^ *kVp*	*120/130* ^a^ *kVp*	*135* ^b^ */140 kVp*
GE	−124 (4.2)	−104 (2.7)	−92 (2.9)	−84 (3.0)
Siemens	−122 (2.4)	−103 (2.5)	−93 (3.0)	−86 (3.2)
Siemens SPECT‐CT	−127 (‐)	−103 (‐)	−95 (‐)	−
Toshiba	−138 (‐)	−112 (‐)	−100 (‐)	−94 (‐)

a The available kVp values on the Siemens SPECT‐CT scanners are 80, 110, and 130 kVp.

b The Toshiba scanner uses 135 kVp instead of 140.

**Table 7:** Acrylic CT number measurements in HU. Data are from 24 GE, 7 Siemens, 3 Siemens SPECT‐CT, and 1 Toshiba scanners. For each entry, the first value is the median and the second value (in parentheses) is the interquartile range

	*80 kVp*	*100/110* ^a^ *kVp*	*120/130* ^a^ *kVp*	*135* ^b^ */140 kVp*
GE	98 (8.1)	114 (5.3)	123 (5.2)	129 (4.3)
Siemens	111 (7.1)	125 (6.3)	132 (5.3)	137 (4.9)
Siemens SPECT‐CT	103 (‐)	121 (‐)	127 (‐)	−
Toshiba	109 (‐)	121 (‐)	125 (‐)	127 (‐)

a The available kVp values on the Siemens SPECT‐CT scanners are 80, 110, and 130 kVp.

b The Toshiba scanner uses 135 kVp instead of 140.

**Table 8:** “Bone” CT number measurements in HU. Data are from 24 GE, 7 Siemens, 3 Siemens SPECT‐CT, and 1 Toshiba scanners. For each entry, the first value is the median and the second value (in parentheses) is the interquartile range

	*80 kVp*	*100/110* ^a^ *kVp*	*120/130* ^a^ *kVp*	*135* ^b^ */140 kVp*
GE	1326 (18)	1121(18)	988 (20)	907(15)
Siemens	1192 (17)	989(16)	880 (16)	821 (28)
Siemens SPECT‐CT	1216 (‐)	961 (‐)	873 (‐)	−
Toshiba	945 (‐)	902 (‐)	933 (‐)	949 (‐)

a The available kVp values on the Siemens SPECT‐CT scanners are 80, 110, and 130 kVp.

b The Toshiba scanner uses 135 kVp instead of 140.

### C. Solid water

Table 4 summarizes the solid water results. The CT numbers are noticeably higher on the GE scanners than on the others. On the Siemens and Toshiba scanners, all values are between −5and4HU. On GE scanners, in contrast, values over 5 HU are common at 120 and 140 kVp (there is a slight trend upward with kVp). Most of the GE scanners have solid water values between 5 and 7 HU at one or more kVps but, on the subsequent manufacturer water phantom scan, all of those scanners were within tolerance (±3HU). The only scanner (mentioned above) which failed the water phantom test had exceptionally high solid water values of 10 to 13 HU. Comparisons between different models of GE scanners are limited by the small numbers of most models. One difference noticed is that the five “older” units (BrightSpeed, LightSpeed Ultra and Pro models) do not reach values as high as most of the newer units; the highest value seen on these five units is only 5.2 HU.

**Figure 2:**
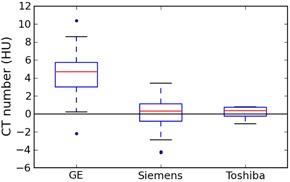
Box plot of solid water values for all kVps, grouped by manufacturer. Whiskers are the highest/lowest points within 1.5*IQR of the upper/lower quartiles; dots are outliers. This and other box plots are produced using the matplotlib library for Python (http://matplotlib.org).

Figure 2 shows the values for all kVps combined, grouped by manufacturer to illustrate the higher values on GE scanners.

### D. Air

Table 5 summarizes the results for air, measured inside and outside the phantom. On GE scanners most values are between −970and−980HU inside the phantom, but outside the values are lower, with a median of about −990HU at each kVp. In both cases there are several outlier values up to about 20 HU away from the median. The Toshiba scanner also has values between −970and−980HU inside the phantom, and lower values outside. In contrast, the Siemens median values are between −990and−1000HU at each kVp, both inside and outside the phantom. The Siemens values contain several outliers (mostly from two of the scanners), which result in large interquartile ranges inside the phantom; outside the phantom there are fewer outliers, and the IQR and variance are smaller.

**Figure 3:**
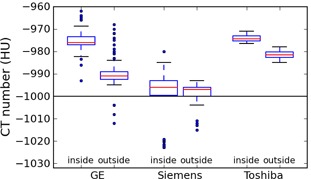
Box plot of air values for all kVps, grouped by manufacturer. For each manufacturer, separate entries are shown for air measured inside the phantom and air measured outside. Whiskers are the highest/lowest points within 1.5*IQR of the upper/lower quartiles; dots are outliers.

Figure 3 shows the values for air inside and outside the phantom, for all kVps combined, grouped by manufacturer to illustrate the higher values on GE and Toshiba scanners.

Four scanners showed unusually high or low air CT numbers and were investigated further. Two were GE scanners (both LightSpeed Pro models) which had high values around −965HU inside the phantom at all kVps. Two were Siemens scanners (both Sensation models) which had low values around −1020HU inside the phantom at all kVps. All four were given a CT number recalibration. However, when the CT numbers were remeasured, the values had not changed significantly (less than 5 HU in all cases). This suggests that the cause of the unusual CT values on these scanners is not miscalibration, but something specific to the scanner — for example, the version of the image reconstruction software. Scanner‐specific issues such as this will be taken into account in our QC program.

### E. Polyethylene and acrylic

Tables 6 and 7 summarize the results for polyethylene and acrylic. Both materials show a clear trend of increasing CT number with kVp on all scanners. For polyethylene, the GE and Siemens values are not significantly different (t‐test p‐value>0.05 at each kVp), while the Toshiba values are lower. For acrylic, the Siemens values are about 10 HU higher than the GE values, which is significant (p‐value<0.01 at each kVp), while the Toshiba values are generally in‐between. The three SPECT‐CT scanners all have similar values, which are about 5–8 HU lower than the median of the other Siemens scanners (using interpolation to compare the values for 110 and 130 kVp).

4, 5 show the values for these two materials, for all manufacturers combined, grouped by kVp to illustrate the kVp dependence.

**Figure 4:**
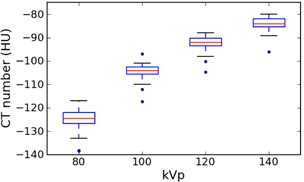
Box plot of polyethylene values for all manufacturers, grouped by kVp. Whiskers are the highest/lowest points within 1.5*IQR of the upper/lower quartiles; dots are outliers.

**Figure 5:**
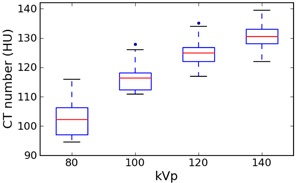
Box plot of acrylic values for all manufacturers, grouped by kVp. Whiskers are the highest/lowest points within 1.5*IQR of the upper/lower quartiles; dots are outliers.

### F. “Bone”

Table 8 summarizes the results for “bone”, which shows clear differences between manufacturers and a decrease of CT number with kVp. The Toshiba scanner is an exception, displaying no definite trend. There is more variation in values between scanners than with other materials, which is reflected in the relatively large interquartile ranges. Siemens values are generally lower than GE values, with the exception of one Siemens scanner with very high values (see below). If this scanner's values are not counted, then the Siemens values are significantly lower than the GE values (*t*‐test p‐value<0.01 at each kVp). The three SPECT‐CT scanners have higher values than other Siemens units (using interpolation to compare the values for 110 and 130 kVp).

**Figure 6:**
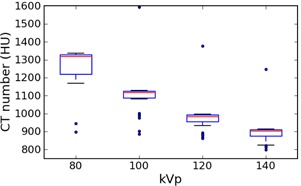
Box plot of “bone” values for all manufacturers, grouped by kVp. Whiskers are the highest/lowest points within 1.5*IQR of the upper/lower quartiles; dots are outliers. One outlier point at 80 kVp, 1937 HU is omitted to allow a smaller scale.

Figure 6 shows the values for “bone”, grouped by kVp; scanners from all manufacturers have been combined for legibility.

The Siemens scanner with very high “bone” values is also one of the two with unusually low air values. The “bone” values range from 1937 HU at 80 kVp to 1248 at 140 kVp: hundreds of HU higher than any other scanner. As mentioned above, this scanner was recalibrated and the air values did not change significantly. Neither did the “bone” values, which changed by less than 20 HU at each kVp. As with the air, the “bone” results on this scanner seem to be caused by a scanner‐specific effect rather than miscalibration. As an additional test on this scanner, a second phantom scan was performed using a body scan instead of a head scan (all other parameters were the same except the DFOV was increased to 36 cm). In the body scan, the CT numbers were much closer to other scanners, between −996and−1001HU for air, and from 1207 HU at 80 kVp to 830 HU at 120 kVp for “bone”.

## IV. DISCUSSION

Comparing the CT numbers of each material between the two ACR phantoms used, the differences are minimal given the acceptable tolerances recommend by the ACR (see Table 2). Differences between the measured CT numbers are much less than the magnitude of the acceptable ranges for each material. The authors believe that the small variations in CT number between the two phantoms can be attributed to manufacturing tolerances on material properties and statistical variations between repeated scans. When devising local tolerances on the CT number of each material for a QC program, it is important to take into account these sources of variation.

Comparing measured CT numbers among our full complement of scanners has shown significant differences in values for solid water, air, and “bone” between GE and Siemens scanners. There are various possible systematic causes of these differences, including differences in beam filtration and in beam‐hardening corrections during image reconstruction. However, for the purposes of quality control, the main goal is to not to explain these differences but to account for them when testing. Therefore, we have used our results to define a set of tolerance ranges for CT numbers of each material, which reflect their observed kVp and manufacturer dependence. The ranges have been set empirically, based on the observed spread of values among scanners. The size of each range attempts to balance the sensitivity and specificity of the QC test. If a range is too large, changes in performance will not be detected; too small, and some scanners with adequate clinical performance will fail the QC test. It is important to note that the ranges may not be applicable if QC scans are performed with certain scan parameters changed (e.g., a body scan instead of a head scan).

Since only one Toshiba scanner was tested, there is insufficient data to define ranges of expected values here specifically for this manufacturer. The same is true for the three Siemens SPECT‐CT scanners. It is interesting to note that the solid water values measured on these four units were all well within the original tolerance range of −5to5HU, and the air values were all within the ACR recommended range of −1005to−970HU. For the other three materials, the observed differences between these four scanners and the larger groups of GE and Siemens scanners suggest that it is not appropriate to group them with the rest when selecting ranges. An alternative would be to compare regular QC measurements to baseline values measured at acceptance.

For solid water, the defined range is −5to+7HU for GE scanners and −5to+5HU for Siemens. In our QC program from now on, a result outside this range at any kVp will require rescanning using the manufacturer‐supplied water phantom. The extended range for GE reflects the higher CT numbers observed in the results above. With this tolerance, in the results above only six of 25 GE scanners (including the one which did require recalibration) and none of the Siemens scanners would require rescanning with the water phantom.

For air, the first decision is whether to measure inside or outside the phantom. Measuring outside generally gives values that are closer to the ideal value of −1000HU. However, measuring air inside the phantom is more clinically relevant, as well as being more consistent with how the other materials are measured. So the tolerance ranges are defined for air measured inside the phantom. Different ranges have been selected for GE and Siemens scanners, which both differ from the ACR‐recommended range. For GE scanners, the range is −1005to−965HU; this is similar to the ACR range (see Table 2) but with the upper limit extended by 5 HU. Values outside this range will require investigation and/or recalibration. Only two of 24 GE scanners in the results above are outside this range (the two which were investigated and recalibrated, as described above). If the upper limit was left at the ACR‐recommended value of −970HU, an additional five scanners would be flagged for recalibration, leading to unnecessary scanner downtime. For Siemens scanners, since no values above −980HU were measured, the selected tolerance range is −1005to−980HU. Two of the ten Siemens scanners above are outside this range (again, the two which were investigated).

For polyethylene and acrylic, the values do not systematically differ between GE and Siemens by more than about 10 HU. So, a reasonably narrow tolerance range can be defined which will cover scanners from both manufacturers. This is easier to use than separate ranges for each manufacturer. However a separate range should be defined for each kVp, given the significant kVp dependence of the measured values. To define the range at each kVp, first the maximum and minimum values were checked and discarded as outliers if they differed considerably from the next value. Then, the remaining maximum (minimum) was rounded up (down) to the nearest 5 HU. The resulting ranges are shown in Table 9. For polyethylene, only one of 24 GE and one of seven Siemens non‐SPECT‐CT scanners in the results above would be flagged using these ranges (both of these are scanners that were also flagged for air). No scanners would be flagged for acrylic.

For “bone”, the GE and Siemens values differ enough that it is worth defining separate ranges for these scanners. The ranges are chosen using the same procedure as for polyethylene and acrylic, except rounding is done to the nearest 10 HU. With these ranges, no GE and two of seven Siemens non‐SPECT‐CT scanners in the results above would be flagged (both Sensation models, one of which is also flagged for air and polyethylene).

Finally, as these results demonstrate a clear dependence on kVp and scanner make, clinicians are advised to consider the HU shift that will result when using lower kVp protocols for dose reduction purposes. Clinical studies should be conducted to evaluate such shifts on a scanner‐by‐scanner basis to establish clear scanner/kVp dependence for clinical interpretation.

**Table 9:** Defined QC tolerance ranges for CT numbers of each material, based on collected data, taking manufacturer and kVp dependence into account. All CT numbers are in HU. For Siemens SPECT‐CT and Toshiba, not enough scanners were surveyed to allow ranges to be defined

	*80 kVp*	*100 kVp*	*120 kVp*	*140 kVp*
Solid water: GE		−5 to 7		
Solid water: Siemens		−5 to 5		
Air: GE		−1005to−965		
Air: Siemens		−1005to−980		
Polyethylene: GE & Siemens	−135to−115	−115to−100	−100to−85	−90to−75
Acrylic: GE & Siemens	90 to 120	110 to 130	120 to 135	125 to 140
“Bone": GE	1280 to 1340	1080 to 1140	940 to 1000	870 to 920
“Bone": Siemens	1160 to 1210	970 to 1010	860 to 900	790 to 830

## V. CONCLUSIONS

In QC scans of the ACR CT phantom using a standardized protocol, the measured CT numbers of solid water and air have been found to differ significantly between scanners from different manufacturers. The dependence of the CT numbers of polyethylene, acrylic, and “bone” on kVp has also been characterized. Based on these results, kVp‐ and manufacturer‐specific tolerance ranges for CT numbers have been defined in order to improve the effectiveness of the QC tests.
